# Supplementary material for: How many submissions are needed to discover friendly suggested reviewers?
Source: PLoS One. 2023 Apr 13;18(4):e0284212. doi: 10.1371/journal.pone.0284212 (PMC10101443; doi:10.1371/journal.pone.0284212)
Supplement: S1 File — (PDF) [file pone.0284212.s001.pdf]

How many submissions are needed to discover friendly suggested reviewers?

Pedro Pessoa[1,2], Steve Pressé[1,2,3],

1 Center for Biological Physics, Arizona State University, Tempe, AZ, USA
2 Department of Physics, Arizona State University, Tempe, AZ, USA
3 School of Molecular Sciences, Arizona State University, Tempe, AZ, USA

* spresse@asu.edu

Supporting information file 1: Cynical moodel results for different numbers of suggested reviewers

This supplemental information section presents results for the inference in both cynical and quality models from simulated data with different numbers of suggested reviewers per submission — four and five. These are compared to the results with three suggestions as presented in the main text. For the cynical model, Fig. 1 presents the marginal probabilities of classes as a function of the number of submissions. We observe that as the number of suggested reviewers grow, it takes more submissions to classify each individual reviewer. This is consistent with the fact that there is more uncertainty in which reviewer was selected by the editor. Moreover, we found that reaching the correct configuration through MAP classification (Fig. 2) required considerably more submissions, while the posterior entropy (Fig. 3) similarly required more submissions to reduce. We also obtain analogous results quality model. The marginal probability of classes, MAP errors and posterior entropy are presented in Figs. 4, 5), and 6.

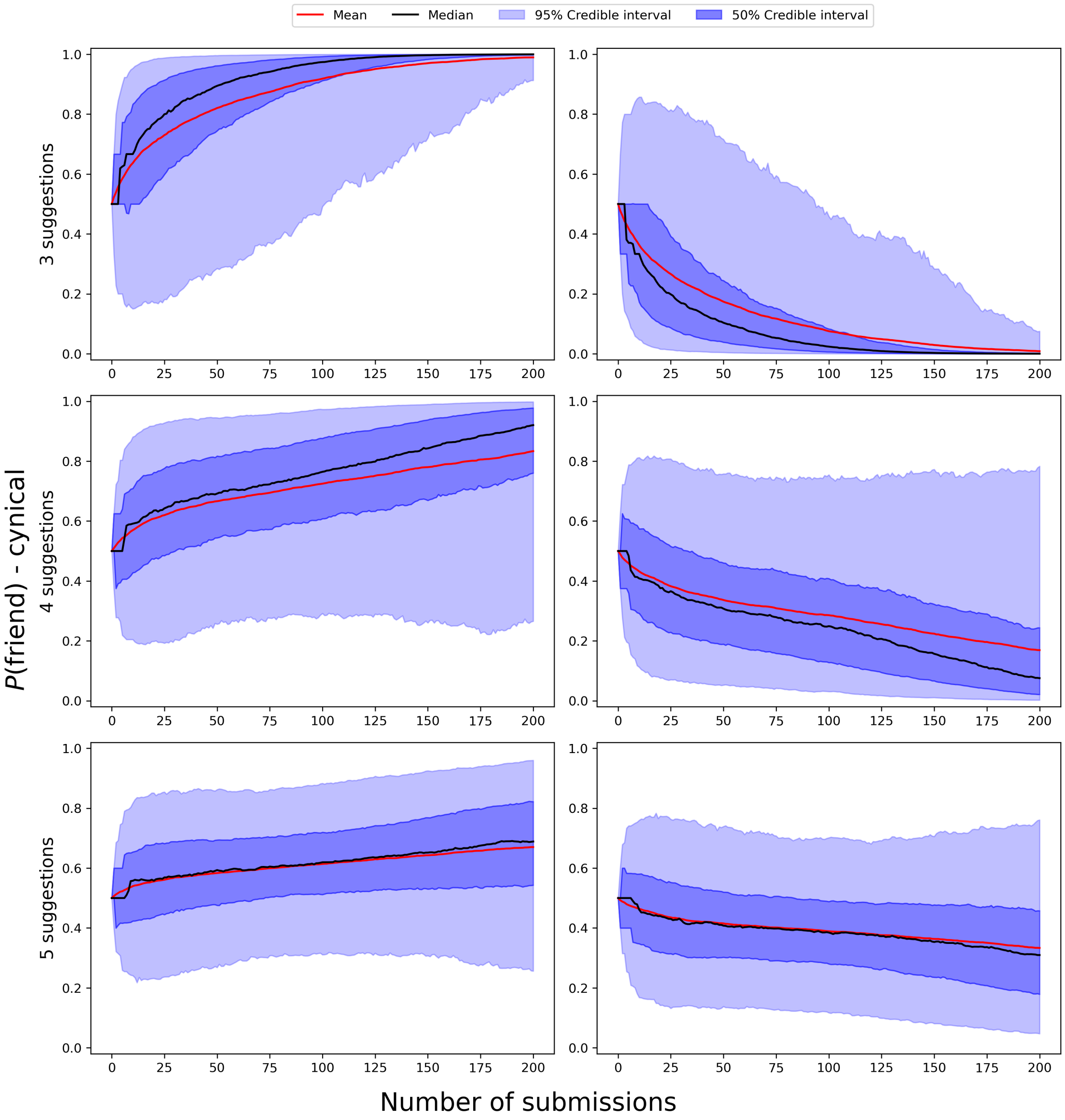

Fig 1. Marginal posterior probability of the cynical model for different number of suggested reviewers. The figure on the left corresponds to friends in the ground truth configuration and on the left right are rivals in the ground truth configuration. In both cases, these trajectories only take into account submissions where the targeted reviewer was suggested. We notice that as the number of suggested reviewers increase, the posterior takes longer to converge, this is expected as with more suggestions, the authors have larger uncertainty on which reviewer was suggested.

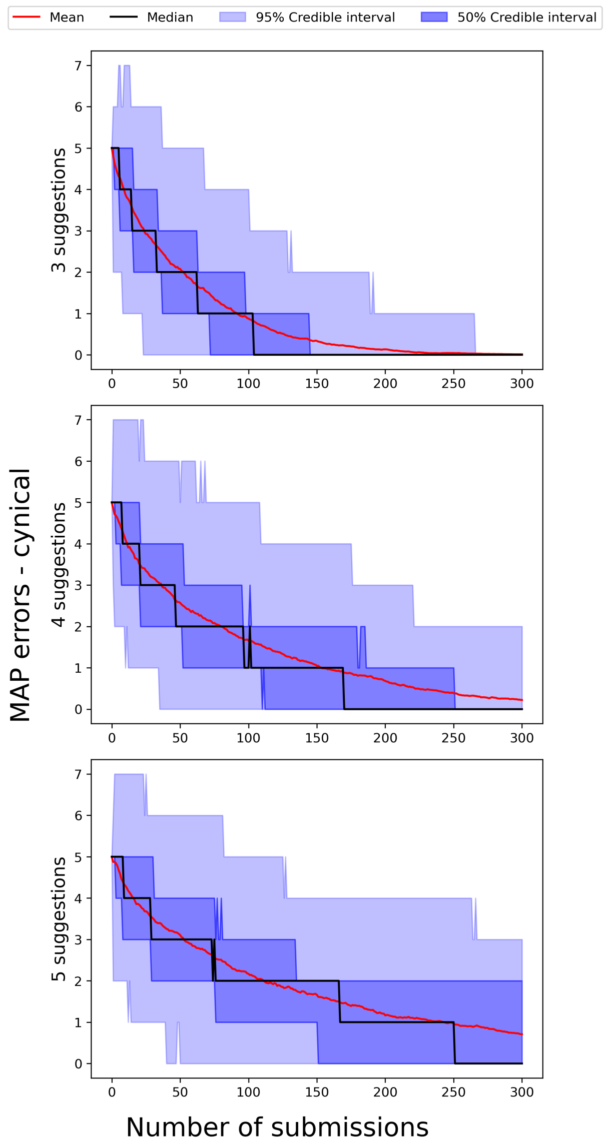

Fig 2. MAP errors of the cynical model for different number of suggested reviewers. We notice that as the number of suggested reviewers increase, more submissions are necessary to discover the correct configuration through MAP.

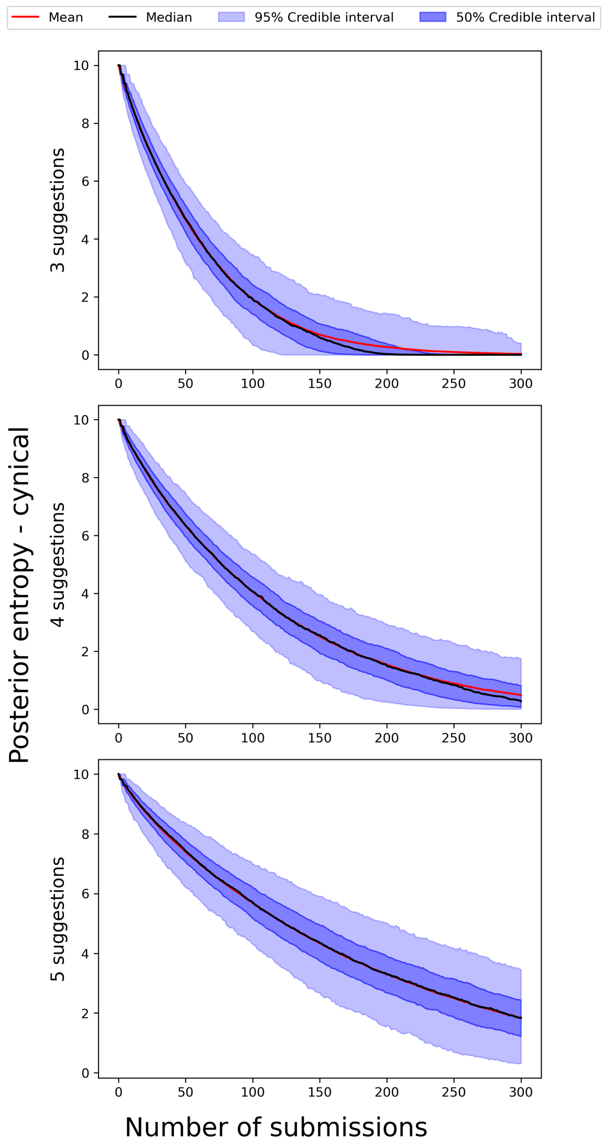

Fig 3. Posterior entropy of the cynical model for different number of suggested reviewers. We notice that as the number of suggested reviewers increase, the entropy is larger for the same number of submissions.

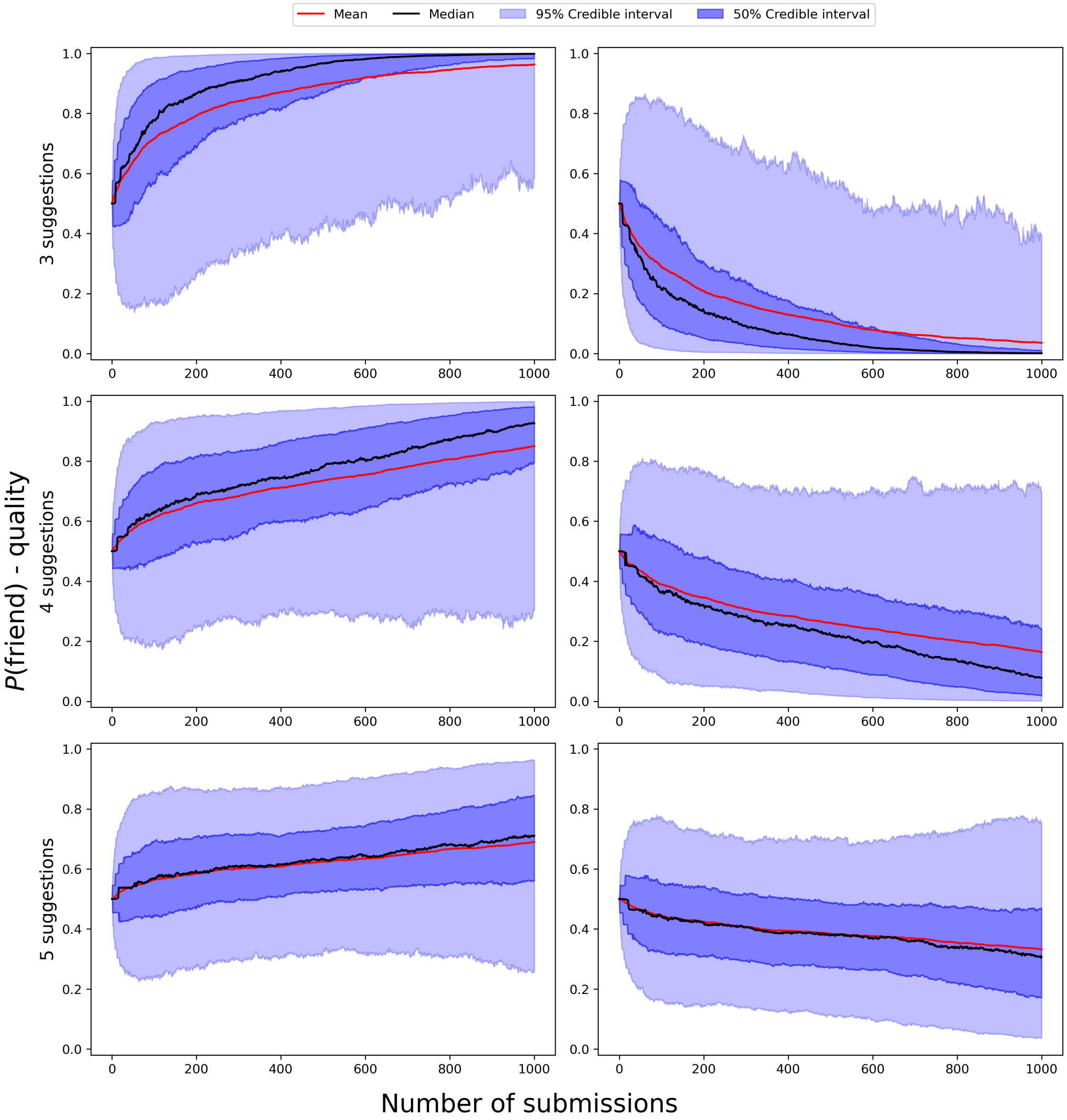

Fig 4. Marginal posterior probability of the quality model for different number of suggested reviewers. Similarly to the cynical model, as the number of suggested reviewers increase the posterior takes longer to converge.

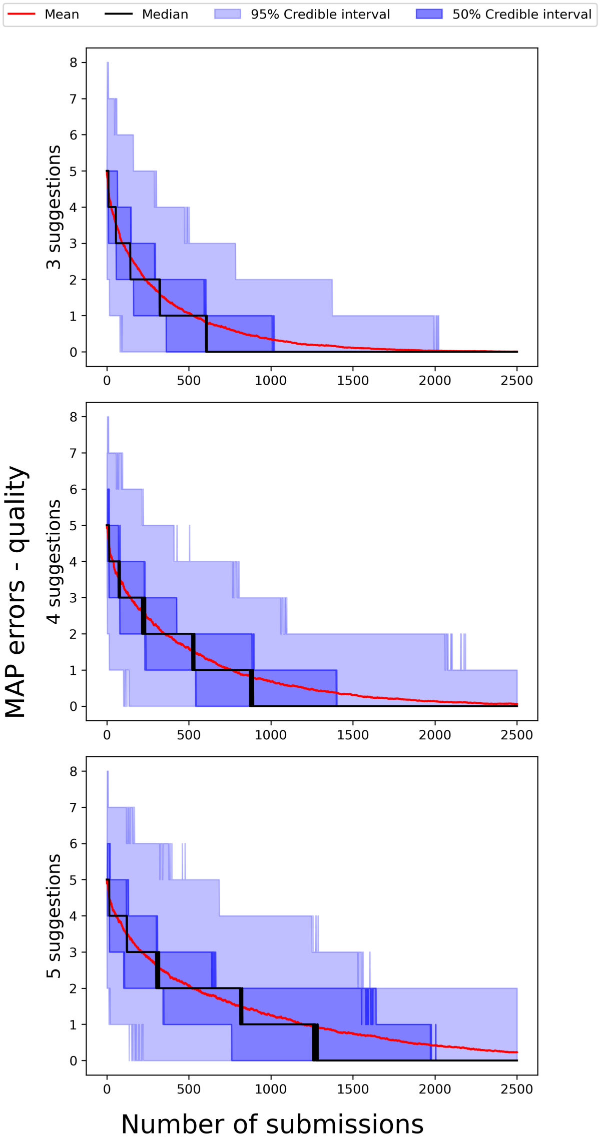

Fig 5. MAP errors of the quality model for different number of suggested reviewers. Similarly to the cynical model, as the number of suggested reviewers increase more submissions are necessary to discover the correct configuration through MAP.

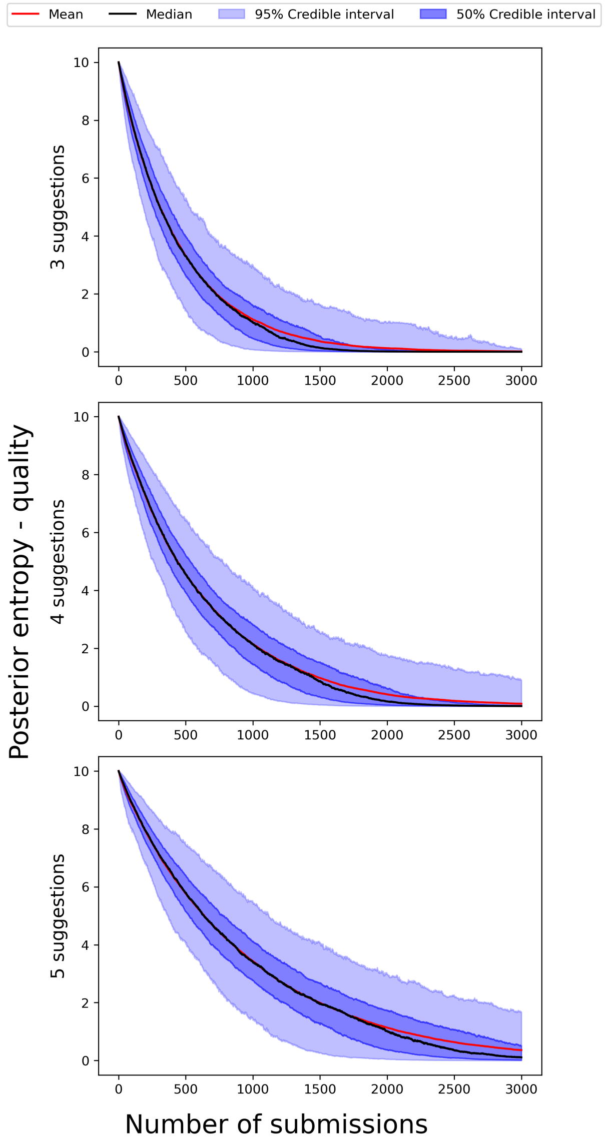

Fig 6. Posterior entropy of the quality model for different number of suggested reviewers. Similarly to the cynical model, as the number of suggested reviewers increase, the entropy is larger for the same number of submissions.
