# Supplementary material for: How many submissions are needed to discover friendly suggested reviewers?
Source: PLoS One. 2023 Apr 13;18(4):e0284212. doi: 10.1371/journal.pone.0284212 (PMC10101443; doi:10.1371/journal.pone.0284212)
Supplement: S2 File — (PDF) [file pone.0284212.s002.pdf]

How many submissions are needed to discover friendly suggested reviewers?

Pedro Pessoa[1,2], Steve Pressé[1,2,3],

1 Center for Biological Physics, Arizona State University, Tempe, AZ, USA
2 Department of Physics, Arizona State University, Tempe, AZ, USA
3 School of Molecular Sciences, Arizona State University, Tempe, AZ, USA

* spresse@asu.edu

Supporting information file 2: Results with a larger ratio of friendly reviewers

This supplemental information section presents results for the inference model from simulated data with a different number of friends — seven and nine out of ten reviewers in $\mathcal{R}$ — in the ground truth. These are contrasted to the result with five friends presented in the main text. Fig. 1 presents marginal probabilities in the cynical model. We observe that the friendly reviewers are classified faster, but the rivals are likely to be mistaken as friends. This qualitative pattern is repeated in the quality model, as it can be seen in Fig. 2. Similarly, we see that the MAP classification, presented in Fig. 3, finds the correct configuration with fewer submissions with a larger ratio of friends. However, for a real classification, the ratio of friends is not known *a priori*. Therefore, it does not necessarily means that an author could classify the reviewers faster.
Entropy, on the other hand, can be calculated directly from the posterior. So it does not require knowing the number of friends *a priori* in order to verify how well classified a set of reviewers is. The posterior entropy for different numbers of friends is presented at Fig. 4. We observe that the median number of friends does not change significantly with the ratio of friends, although the fluctuations are smaller in the cynical model. Regardless of the number of friends in the ground truth configuration, it still takes between 150 and 200 submissions in the cynical model — and around 1500 in the quality model — to correctly classify a set of ten reviewers.
Finally, the number of submissions necessary to reach 95% credibility in the cynical model can be seen in Fig. 5, as the ratio of friends increase the number of submissions necessary decreases reaching approximately 40 when there is 9 out of 10 friends. Similarly, as the number of friends in the ground truth increases from 5 to 9, it is needed a little less than 200 submissions to obtain the same credibility in the quality model (Fig. 6).

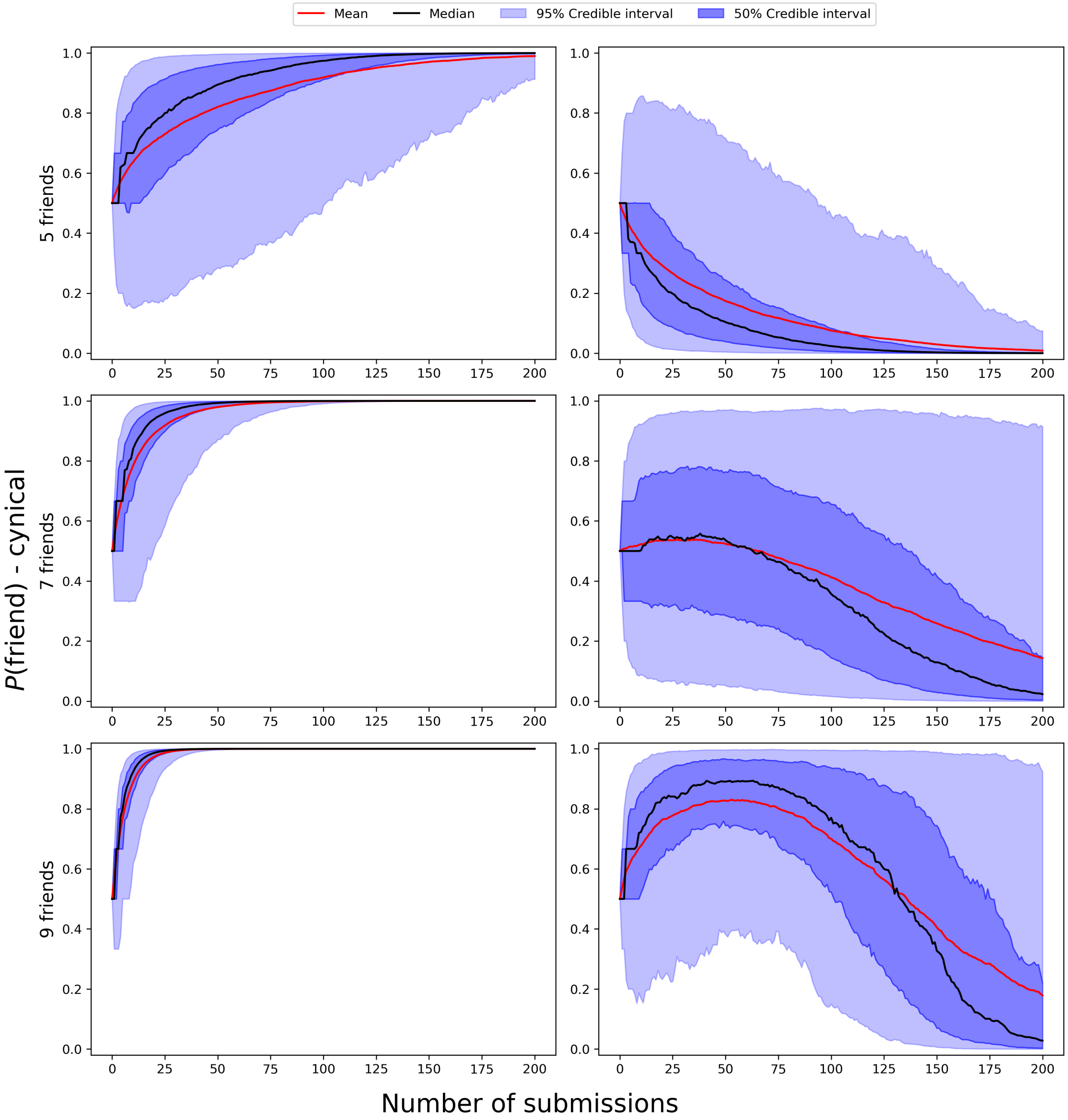

Fig 1. Marginal posterior probability for the cynical model. The figure on the left corresponds to friends in the ground truth configuration and on the left right are rivals in the ground truth configuration. In both cases, these trajectories only take into account submissions where the targeted reviewer was suggested. We notice that as the ratio of friends increases, friends are classified with fewer submissions, but rivals are more likely to be misclassified.

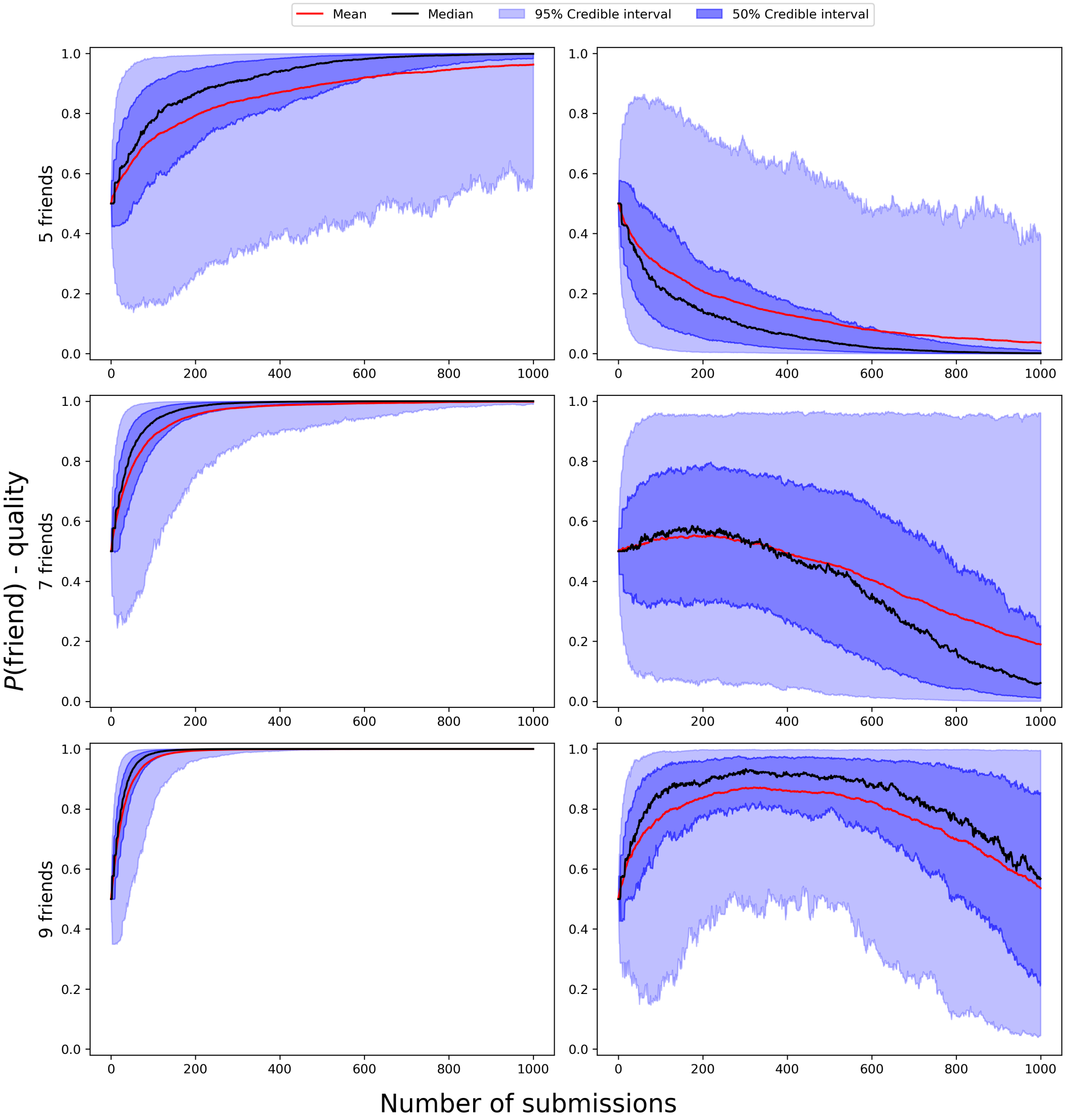

Fig 2. Marginal posterior probability for the quality model. The figure on the left corresponds to friends in the ground truth configuration and on the right are rivals in the ground truth configuration. The pattern is similar to the one in the cynical model, except for the significantly larger number of submissions required.

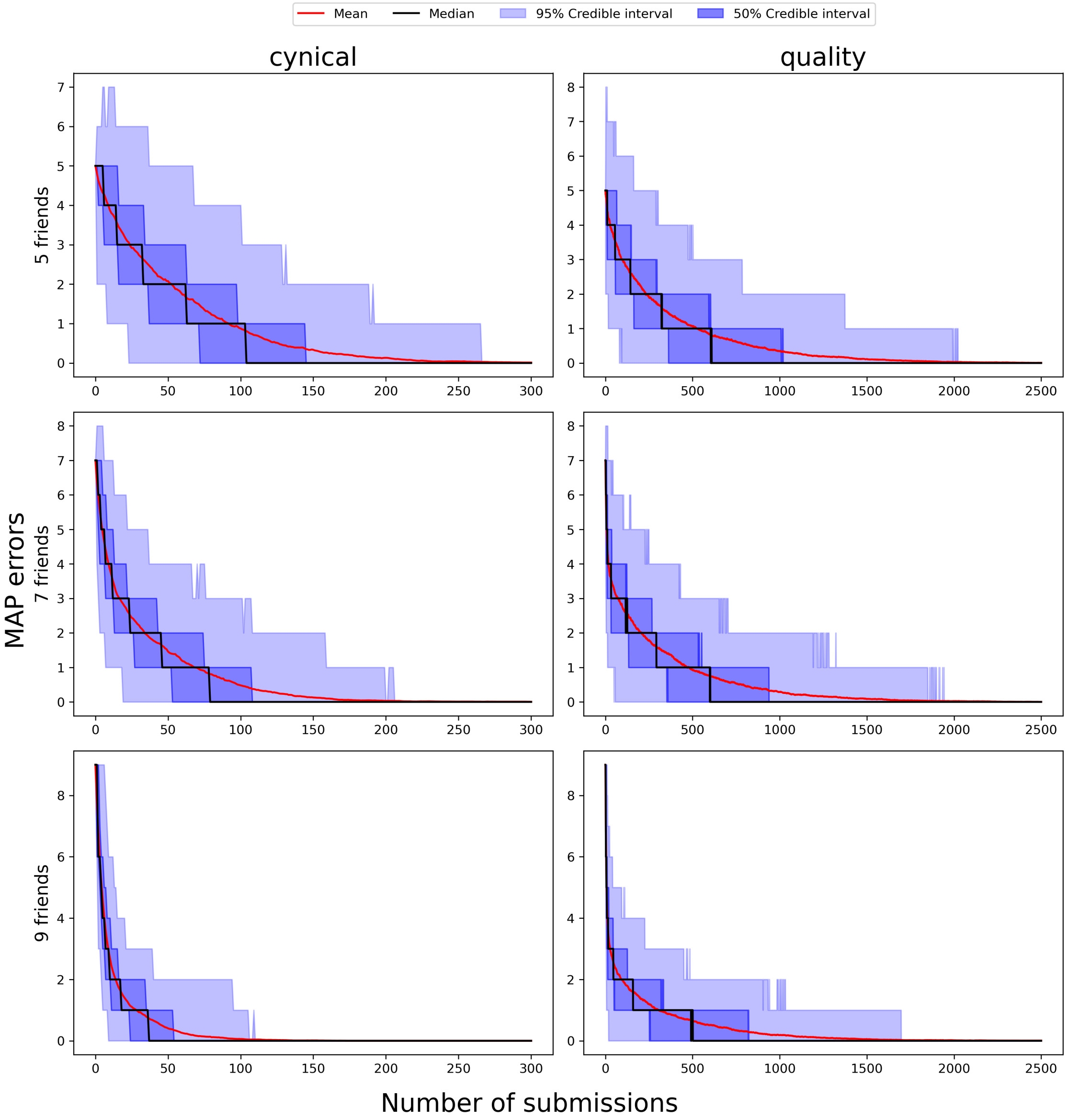

Fig 3. MAP errors for different ratios of friends in the ground truth configuration. We notice that for a greater ratio of friends, it takes fewer submissions for the MAP configuration to match the ground truth configuration. However, this would not necessarily help in classification, as the author does not *a priori* know the number of friends in the set of suggested reviewers.

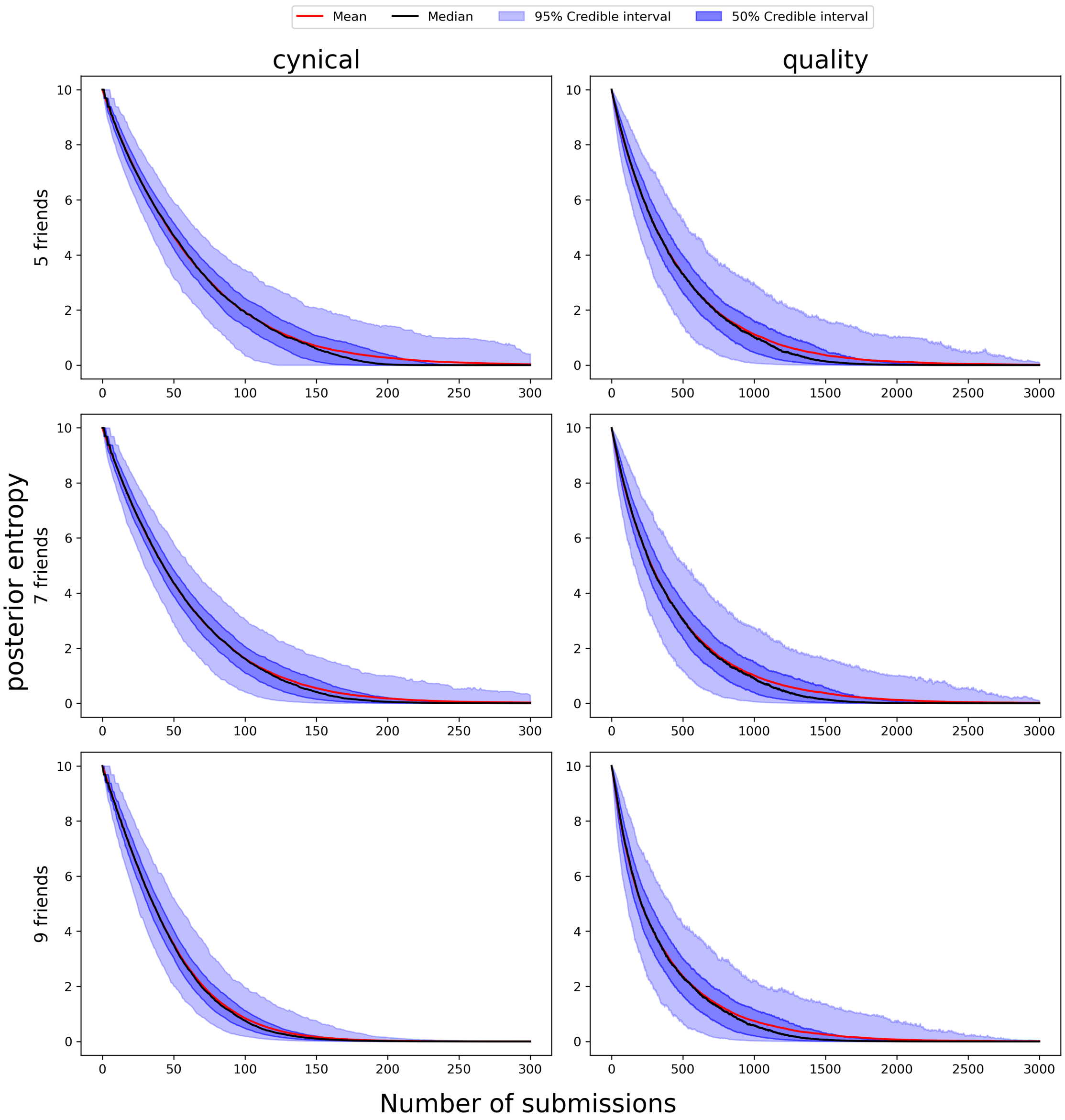

Fig 4. Posterior's entropy for different ratios of friends in the ground truth configuration. The posterior visibly changes with the number of friends in the cynical model. However it is still necessary around 150 submissions to fully classify the reviewers. Nevertheless, the 50% and 95% credible intervals are closer to the median for higher ratio of friends — indicating smaller fluctuations. In the quality model such changes are not clearly visible.

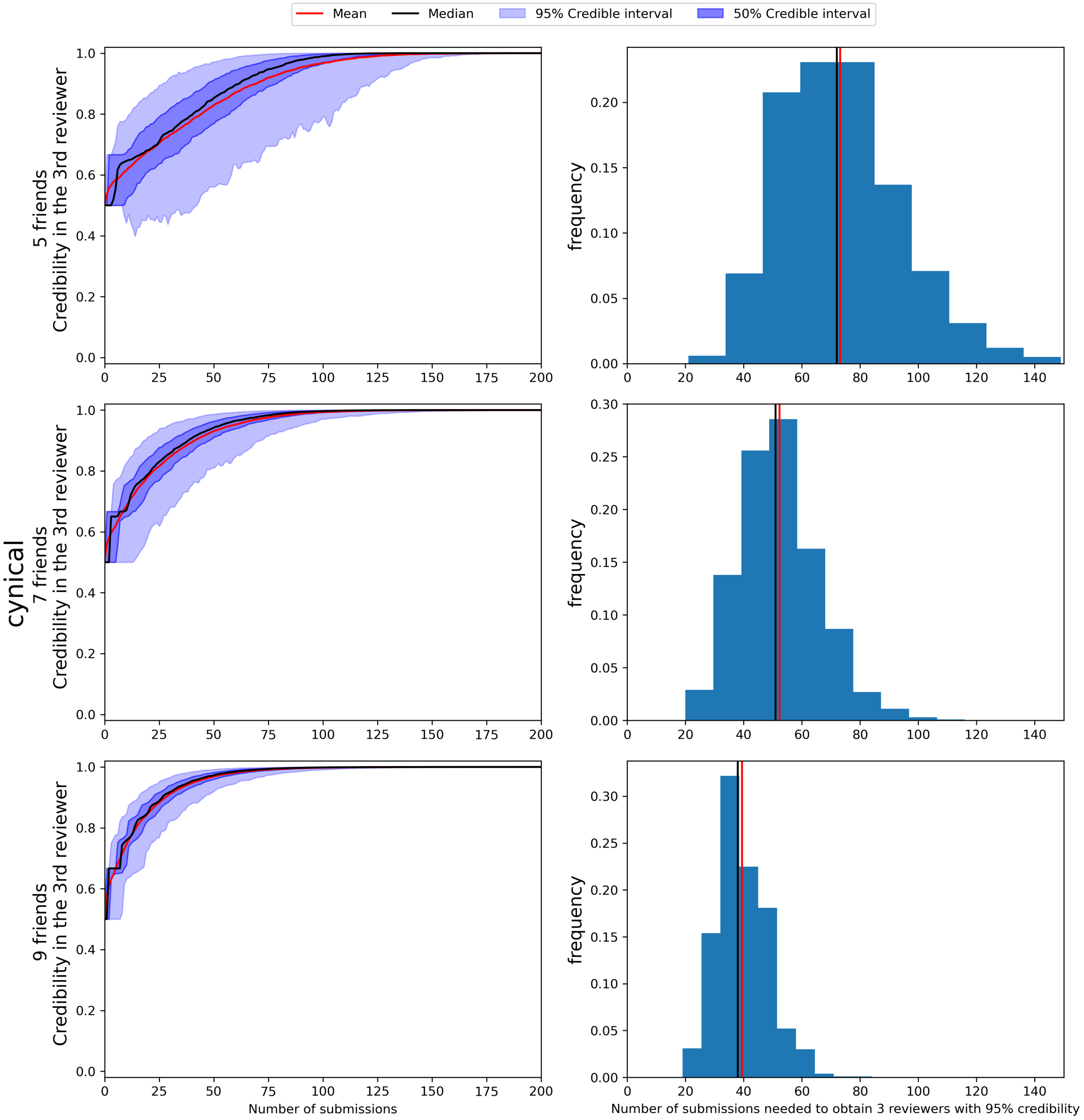

Fig 5. Marginal probability of the third most likely reviewer to be friendly (left) and number of submissions necessary to obtain 95% credibility for three suggested reviewers (right) in the cynical model. The posterior visibly changes with the number of friends. However it is still necessary to have 40 submissions in order to fully classify reviewers.

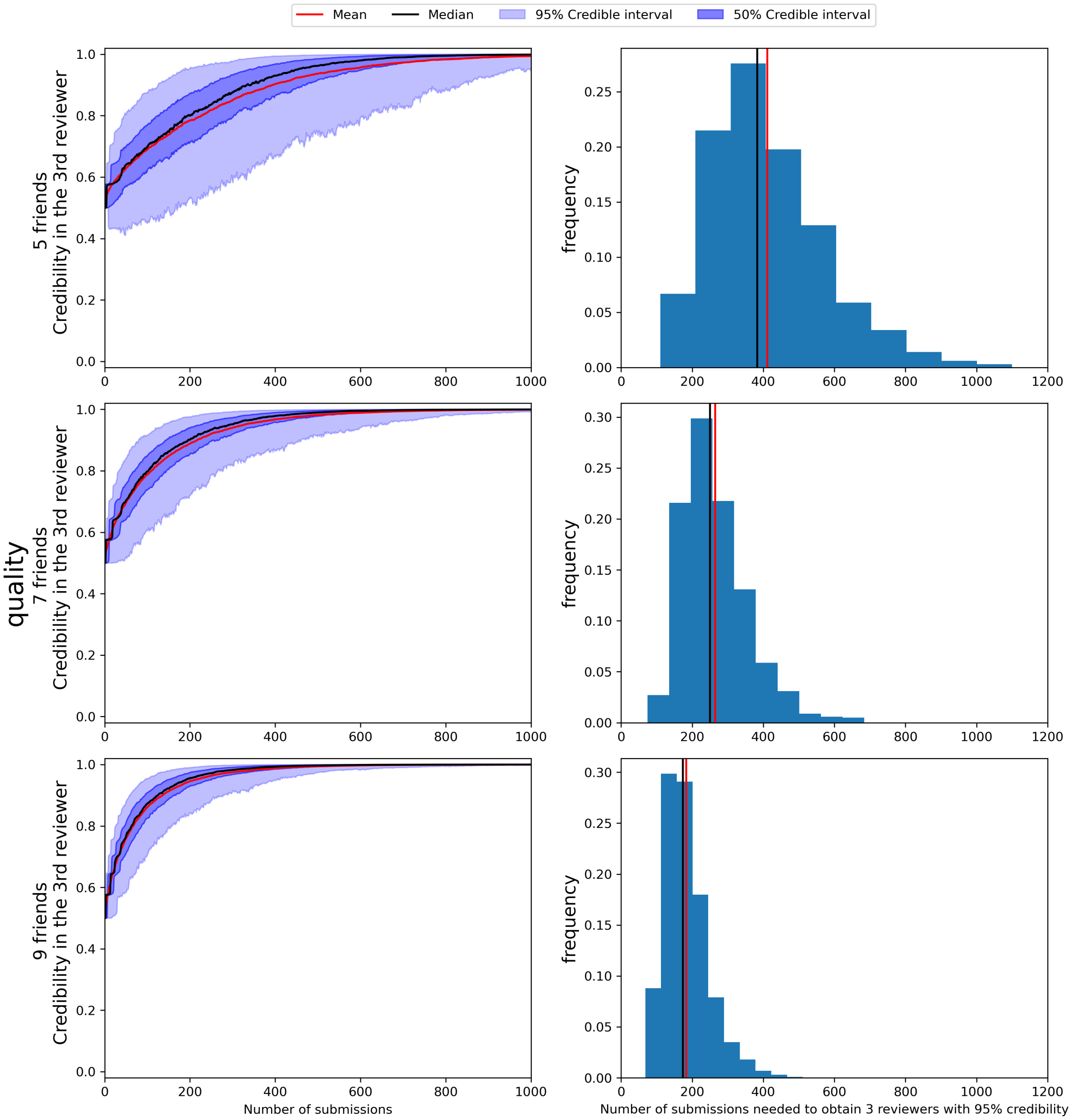

Fig 6. Marginal probability of the third most likely reviewer to be friendly (left) and number of submissions necessary to obtain 95% credibility for three suggested reviewers (right) in the quality model. The posterior visibly changes with the number of friends. However it is still necessary to have around 200 submissions to fully classify the reviewers, a number rather large for all but the most prolific scientists.
