# Supplementary material for: How many submissions are needed to discover friendly suggested reviewers?
Source: PLoS One. 2023 Apr 13;18(4):e0284212. doi: 10.1371/journal.pone.0284212 (PMC10101443; doi:10.1371/journal.pone.0284212)
Supplement: S3 File — (PDF) [file pone.0284212.s003.pdf]

How many submissions are needed to discover friendly suggested reviewers?

Pedro Pessoa[1,2], Steve Pressé[1,2,3],

1 Center for Biological Physics, Arizona State University, Tempe, AZ, USA
2 Department of Physics, Arizona State University, Tempe, AZ, USA
3 School of Molecular Sciences, Arizona State University, Tempe, AZ, USA

* spresse@asu.edu

Supporting information file 3: Sampling details

In this supplemental information section, we detail the simulation described in Section 2.2 (Simulation). We present sampling equations for a single submission review, this process is repeated M times and each sampled number of positive reviews, a, is assigned a label $\mu \in \{1, 2, \ldots, M\}$. We separate the sampling equations for the cynical and quality models in the two following subsections.

Cynical

The simulation generating data in the cynical model begins with the editor choosing one reviewer, r_1, from the list of suggested reviewers, $\mathcal{S}$, with uniform probability. As done in Section 2.3 in the main text, we use r_1 as the class of the suggested reviewer selected by the editor. If reviewer i is selected, $r_1 = x_i$ where x is the ground truth configuration. The number of positive reports, a is the sum of two terms: the first, χ_{r_1} corresponding to the report written by r_1, hence it will be 1 if $r_1 = \textit{friend}$ and 0 if $r_1 = \textit{rival}$; while the second one, χ_{r_2} corresponding to the report written by r_2, which is equally likely to be 0 or 1. The summary of the relevant sampling equations is

$$r_1 \sim \mathbf{C}\text{ategorical}_{s_{1:|\mathcal{S}|}} \left(\frac{1}{|\mathcal{S}|}, \ldots, \frac{1}{|\mathcal{S}|} \right) \ , \tag{1a}$$

$$\chi_{r_1} = \begin{cases} 0 & \text{if} \quad r_1 = \textit{rival} \\ 1 & \text{if} \quad r_1 = \textit{friend} \end{cases} , \tag{1b}$$

$$\chi_{r_2} \sim \mathbf{C}\text{ategorical}_{0,1} \left(1/2, 1/2 \right) \ , \tag{1c}$$

$$a = \chi_{r_1} + \chi_{r_2} \ ; \tag{1d}$$

where the symbol $\sim$ means "sampled from".

Quality

As mentioned in Section Section 2.2 in the main text, the major difference between the quality and the cynical model is that we need to sample the quality factor q, using a Beta distribution as in Eq. 1 in the main text. As in the cynical model, the number of positive reports is the sum of two terms σ_{r_1} and σ_{r_2} representing the reviewers r_1 and r_2 respectively. In accordance with Table 2 in the main text, if $r_1 = \textit{rival}$, then χ_{r_1} will be 1 — or the report written by r_1 will be positive — with probability q^2 and $\chi_{r_1} = 0$ with probability $1 - q^2$. If $r_1 = \textit{friend}$, we have $\chi_{r_1} = 1$ with probability $q(2 - q)$, $\chi_{r_1} = 0$ and with probability $(1 - q)^2$. Similarly, χ_{r_2} will be 1 with probability q and 0 otherwise. Also, as in the cynical model, the editor selects the reviewer r_1 uniformly from the list of suggested reviewers $\mathcal{S}$. The summary of the relevant

sampling equations is

$$q \sim \mathbf{B}\text{eta}(\alpha, \beta) \tag{2a}$$

$$r_1 \sim \mathbf{C}\text{ategorical}_{s_{1:|\mathcal{S}|}}\left(\frac{1}{|\mathcal{S}|}, \dots, \frac{1}{|\mathcal{S}|}\right) \ , \tag{2b}$$

$$\chi_{r_1} \sim \begin{cases} \mathbf{C}\text{ategorical}_{0,1}(1-q^2, q^2) & \text{if} \quad r_1 = \textit{rival} \\ \mathbf{C}\text{ategorical}_{0,1}((1-q)^2, q(2-q)) & \text{if} \quad r_1 = \textit{friend} \end{cases} \ , \tag{2c}$$

$$\chi_{r_2} \sim \mathbf{C}\text{ategorical}_{0,1}\left(1-q, q\right) \ , \tag{2d}$$

$$a = \chi_{r_1} + \chi_{r_2} \ . \tag{2e}$$
