# Supplementary material for: How many submissions are needed to discover friendly suggested reviewers?
Source: PLoS One. 2023 Apr 13;18(4):e0284212. doi: 10.1371/journal.pone.0284212 (PMC10101443; doi:10.1371/journal.pone.0284212)
Supplement: S4 File — (PDF) [file pone.0284212.s004.pdf]

How many submissions are needed to discover friendly suggested reviewers?

Pedro Pessoa[1,2], Steve Pressé[1,2,3],

1 Center for Biological Physics, Arizona State University, Tempe, AZ, USA
2 Department of Physics, Arizona State University, Tempe, AZ, USA
3 School of Molecular Sciences, Arizona State University, Tempe, AZ, USA

* spresse@asu.edu

Supporting information file 4: Quality results with different parameters

In this supplemental information section, we justify the use of the Beta distribution with parameters $\alpha = 12$ and $\beta = 12$ to present a lower bound on the number of submissions. As mentioned in main text, beta distributions are typical choices for sampling random variables distributed over the interval $(0, 1)$. From Eq.1 in the main text we calculate the expected value and variance of the quality factor, $\langle q \rangle$ and σ_q respectively, obtaining

$$\langle q \rangle = \frac{\alpha}{\alpha+\beta} \quad , \quad \sigma_q = \frac{\alpha\beta}{(\alpha+\beta)^2(\alpha+\beta+1)} \ . \tag{1}$$

Which can be inverted as

$$\alpha = -\langle q \rangle \left(\frac{\langle q \rangle^2 - \langle q \rangle + \sigma_q}{\sigma_q} \right) \quad , \quad \beta = \alpha \left(\frac{1}{\langle q \rangle} - 1 \right) \ . \tag{2}$$

In the main text, we studied a scientist with median papers with small variance — $\alpha = 12$ and $\beta = 12$ implies, from (1), $\langle q \rangle = 1/2$ and $\sigma_q = .01$. Here we will compare this to scientists with overall smaller and bigger qualities — $\langle q \rangle = .25$ and $\langle q \rangle = .75$ respectively — and smaller and bigger variances — $\sigma_q = .05$ and $\sigma_q = .005$ respectively. In Table 1 we obtain the associated values of α and β for this study.

$\langle q \rangle \backslash \sigma_q$	.05	.01	.005
.25	$\alpha = 0.6875$<br>$\beta = 2.0625$	$\alpha = 4.4375$<br>$\beta = 13.3125$	$\alpha = 9.125$<br>$\beta = 27.375$
.5	$\alpha = 2$<br>$\beta = 2$	$\alpha = 12$<br>$\beta = 12$	$\alpha = 24.5$<br>$\beta = 24.5$
.75	$\alpha = 2.0625$<br>$\beta = 0.6875$	$\alpha = 13.3125$<br>$\beta = 4.4375$	$\alpha = 27.375$<br>$\beta = 9.125$

Table 1. Values of α and β obtained from (2) in terms of $\langle q \rangle$ and σ_q for the examples we study in this section.

Fig. 1 presents the MAP errors for each of these values. We observe that, as the values of $\langle q \rangle$ move away from $1/2$, it require more submissions to correctly classify reviewers. Thus, the lower bound is found by a researcher whose submissions are of median quality $\langle q \rangle = 1/2$. These results are confirmed by an equivalent figure plotting the posterior's entropy in Fig. 2.
On the other hand, we also observe (in Figs. 1, 2 and 3) that the smaller the variance, σ_q, the fewer submissions are necessary, indicating that the lower bound is also found for minimal variance in quality, σ_q. Although a scientist with $\sigma_q \to 0$ is not realistic, this can still be simulated in the model — $q_\mu = \langle q \rangle$ for every submission. In Fig. 4 we present a comparison for MAP errors of quality $\sigma_q = 0.01$ and $\sigma_q = 0$. We observe that although the number of submissions necessary is smaller for $\sigma_q = 0$, in the case of $\langle q \rangle = 1/2$ the

difference is not significant — both require a little over 500 submissions. The analogous results for the posterior's entropy — presented in Fig. 5 — shows a bigger difference but is still necessary to have more than 1500 submissions to correctly classify reviewers. Finally, the number of submissions necessary to reach 95% with $\sigma_q = 0$ is presented in Fig. 6. Even in the limit of zero variance, around 300 submissions are necessary to obtain 3 reviewers classified with 95% credibility.

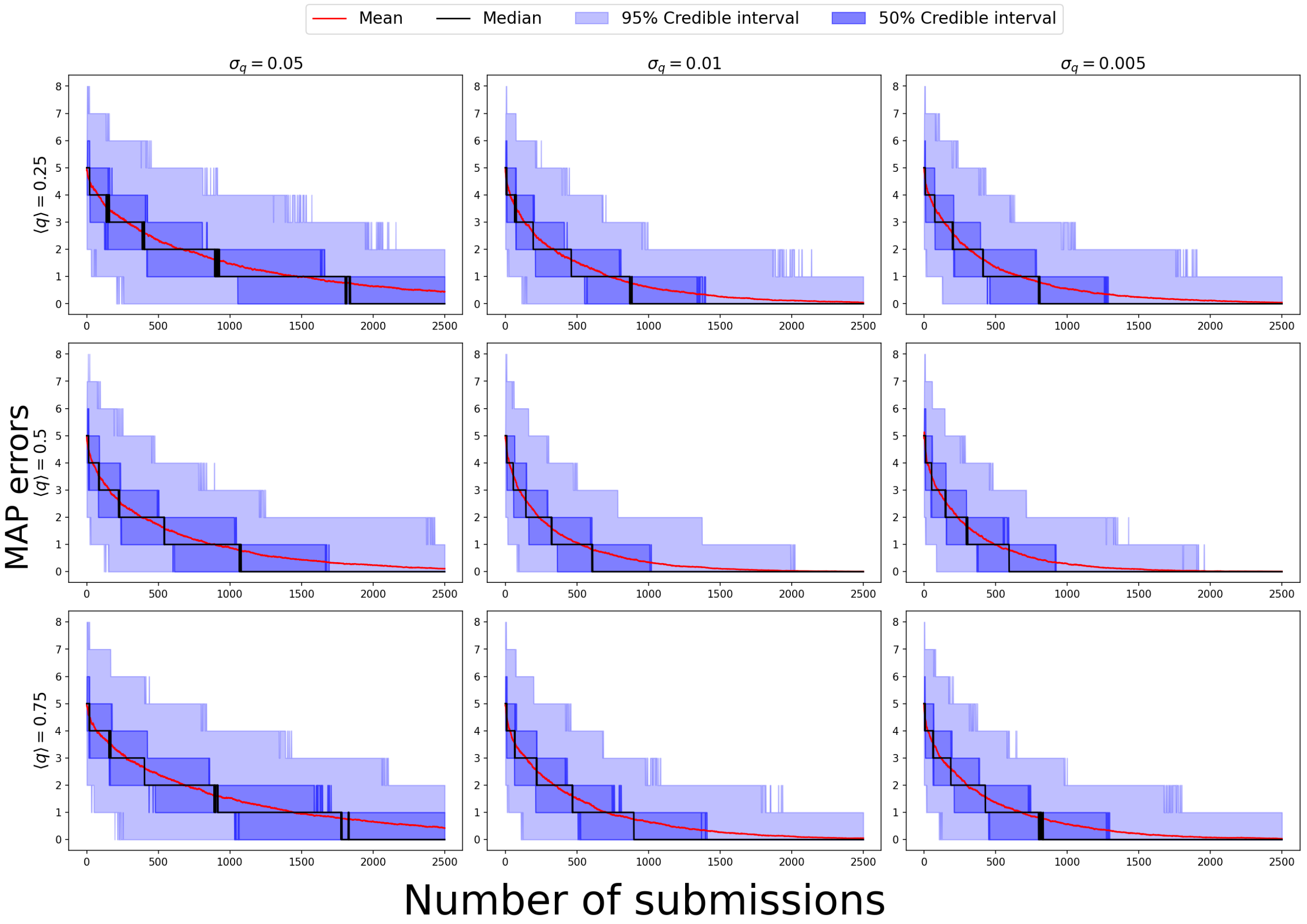

Fig 1. MAP errors obtained for different sampling distributions of quality factors. We notice that the number of submissions necessary to find the correct configuration is smaller for the expected value $\langle q \rangle = .5$ — representing a scientist with overall median quality articles. The number of submissions necessary also decreases as the variance reduces.

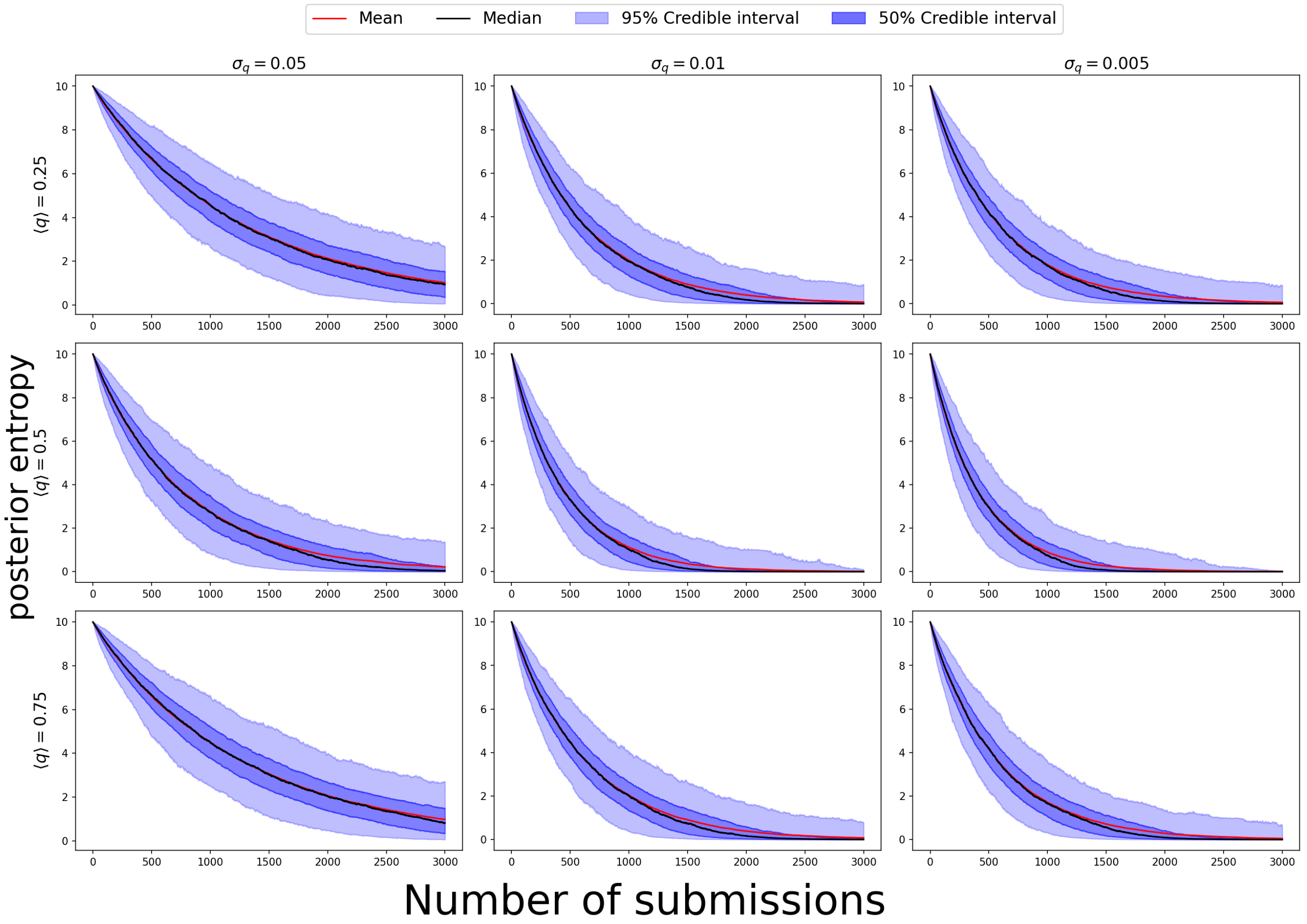

Fig 2. The posterior's entropy obtained for different sampling distributions of quality factors. We notice that the number of submissions necessary to fully classify reviewers is smaller for the expected value $\langle q \rangle = 1/2$ when compared to both larger and smaller expected values. The number of submissions necessary also decreases as the variance reduces.

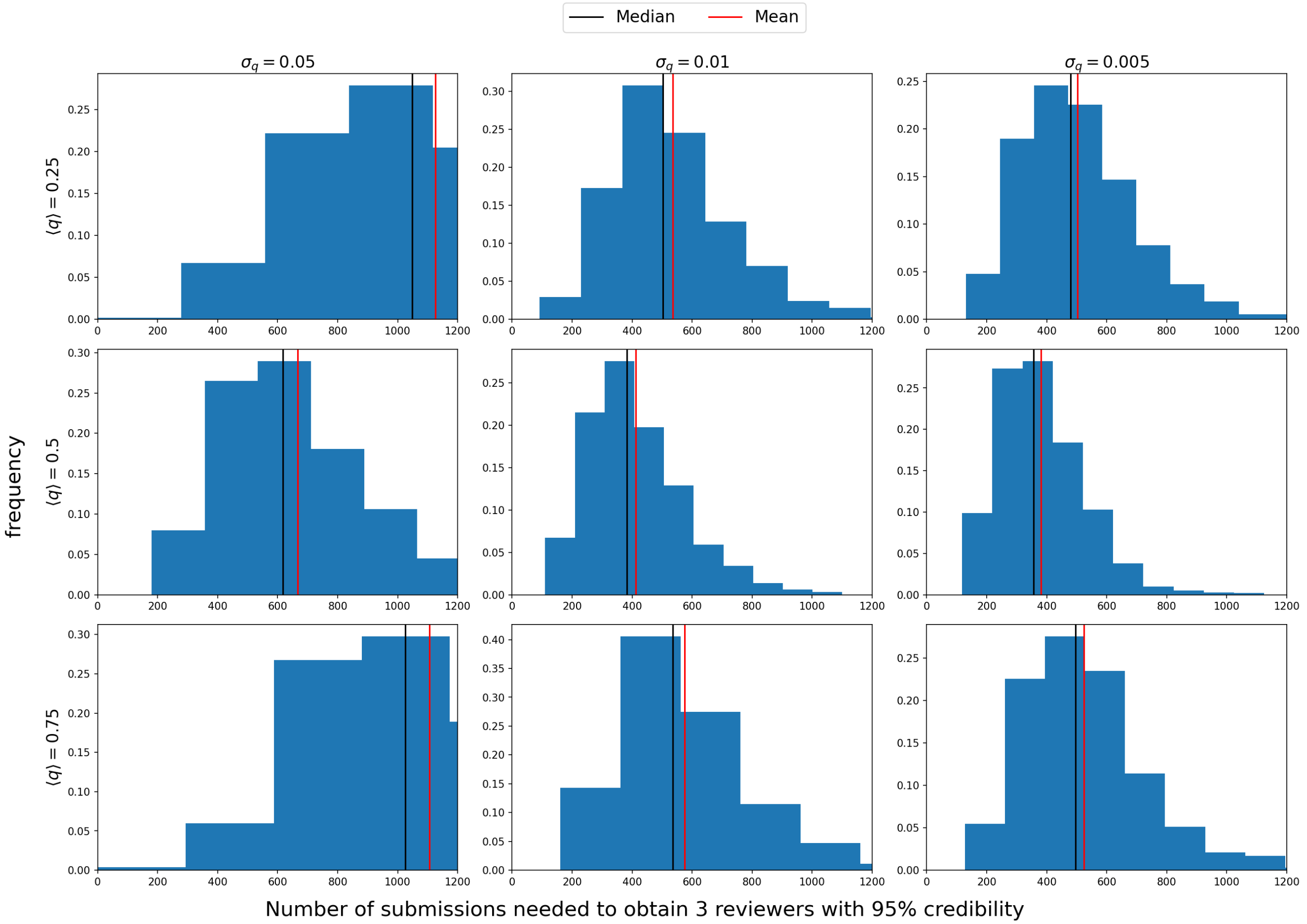

Fig 3. Number of submissions necessary to achieve 95% credibility for three suggested reviewers obtained for different quality factor distributions. Some of these graphs were truncated in order to present better comparison to the central case, $\langle q \rangle = {}^1/_2$. We notice that the number of submissions necessary to classify at least three suggested reviewers is smaller for the expected value $\langle q \rangle = {}^1/_2$ when compared to both larger and smaller expected values. The number of submissions necessary also decreases as the variance reduces.

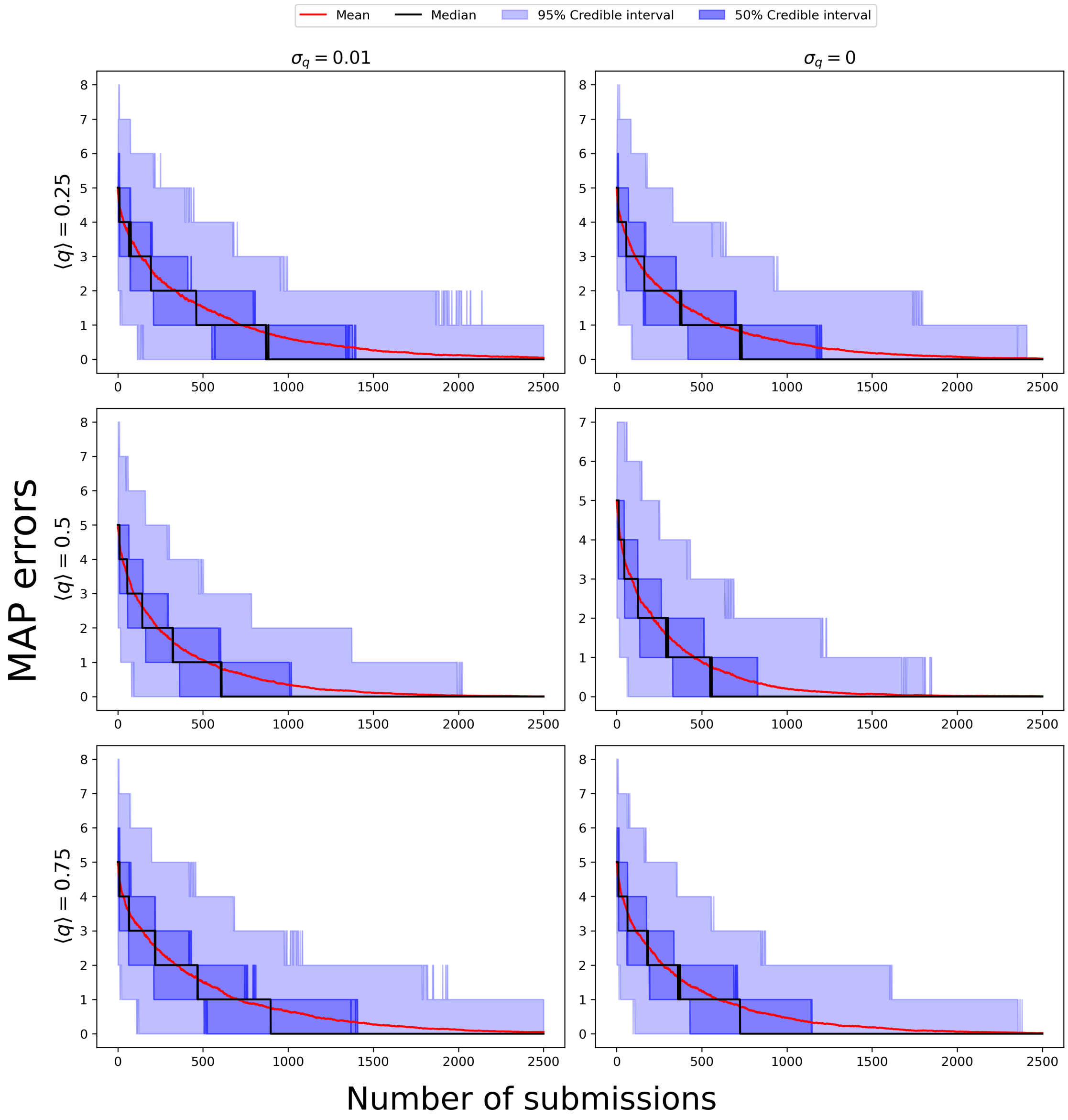

Fig 4. MAP errors comparing the distribution of variance $\sigma_q = 0.01$, as in the main text, to one of zero variance (all sampled quality factor have the same value). Although the number of submissions necessary to find the correct configuration decreases when we change from $\sigma_q = 0.01$ to 0, it does not change dramatically for $\langle q \rangle = 1/2$, in both cases needing more than 500 submissions in the median case. Thus, the overall lower bound is larger than 500 submissions and our choice of $\alpha = \beta = 12$ — or analogously, a Beta distribution with $\langle q \rangle = 1/2$. and $\sigma_q = 0.01$ — as an estimator for the lower bound in a more realistic case.

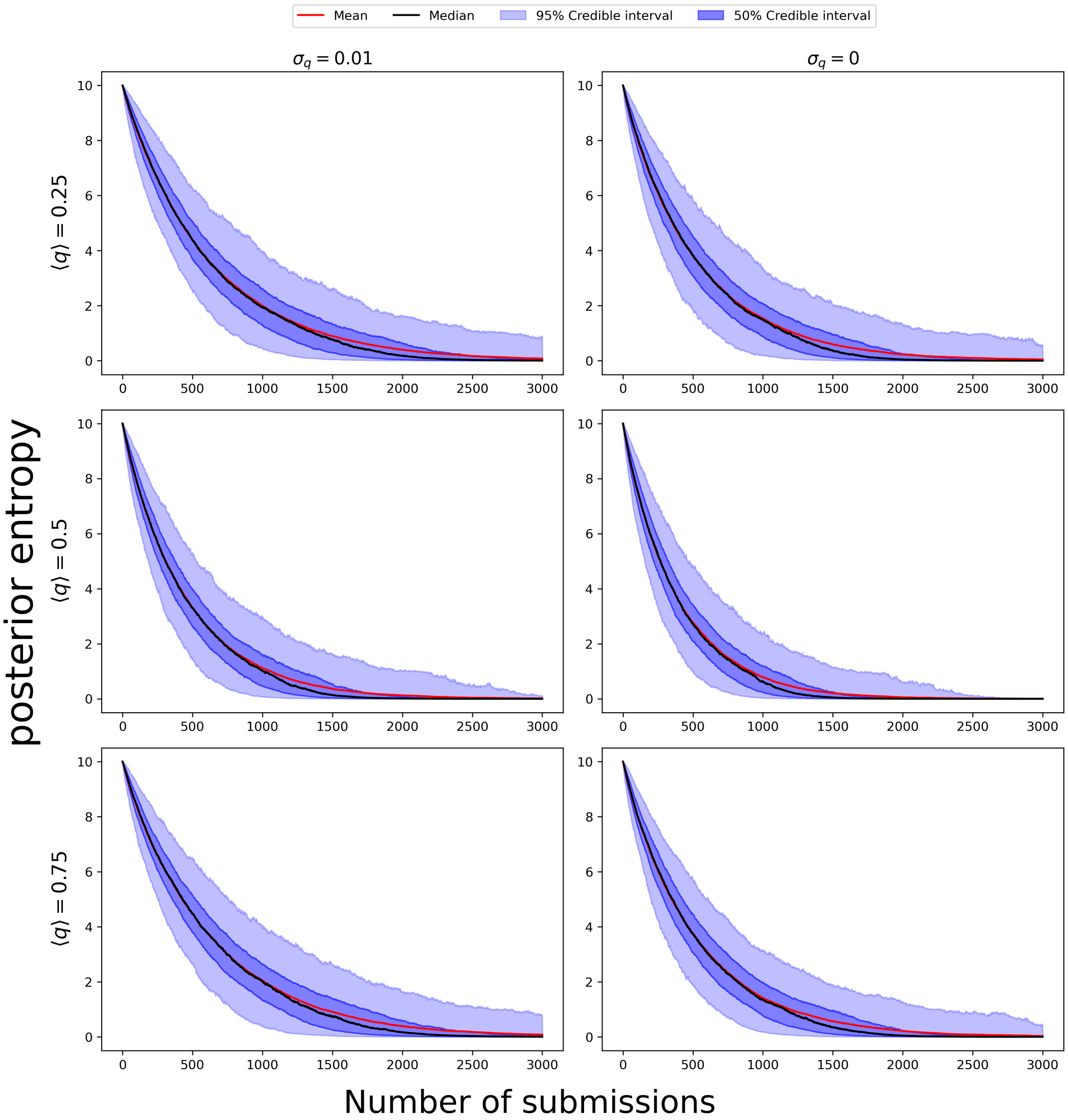

Fig 5. The posterior's entropy comparing the distribution of variance $\sigma_q = 0.01$, as in the main text, to one of zero variance. Similarly to Fig. 4, we notice that the difference is not significant for $\langle q \rangle = {}^1\!/_2$.

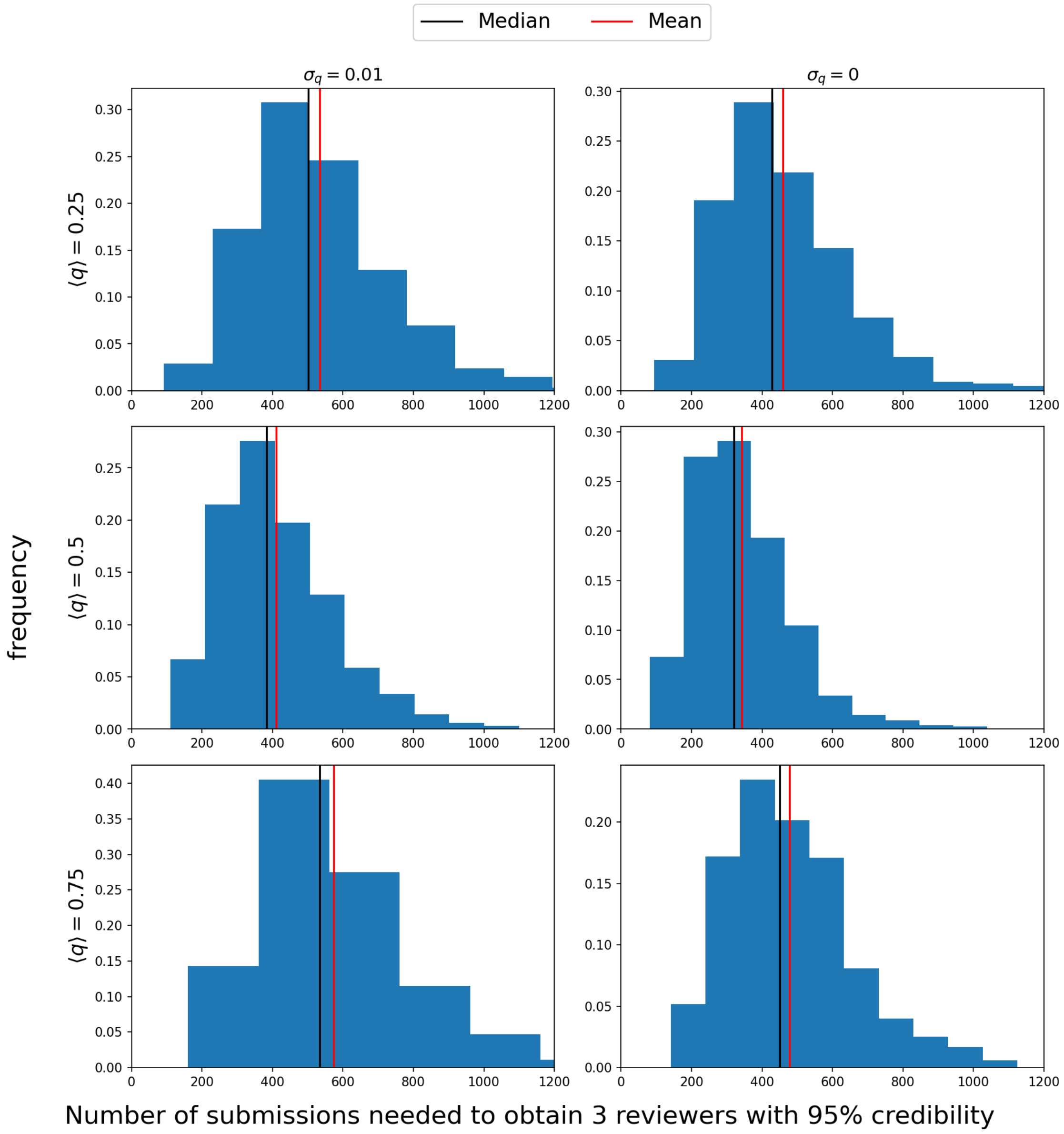

Fig 6. Number of submissions necessary to obtain 95% credibility for three suggested reviewers obtained for different sampling distributions of quality factors. We notice that 300 submissions are still necessary to obtain this degree of credibility even for zero variance.
