# Supplementary material for: How many submissions are needed to discover friendly suggested reviewers?
Source: PLoS One. 2023 Apr 13;18(4):e0284212. doi: 10.1371/journal.pone.0284212 (PMC10101443; doi:10.1371/journal.pone.0284212)
Supplement: S5 File — (PDF) [file pone.0284212.s005.pdf]

How many submissions are needed to discover friendly suggested reviewers?

Pedro Pessoa[1,2], Steve Pressé[1,2,3],

1 Center for Biological Physics, Arizona State University, Tempe, AZ, USA
2 Department of Physics, Arizona State University, Tempe, AZ, USA
3 School of Molecular Sciences, Arizona State University, Tempe, AZ, USA

* spresse@asu.edu

Supporting information file 5: Errors and aggressive strategy for the fourth metric

Here, we delve deeper into the fourth metric discussed in the main text by providing a detailed analysis of the number of misclassifications occurring when attempting to achieve 95% credibility among the top three reviewers, meaning how often at least one of the reviewers between the top three with higher than 95% credibility are rivals in the ground truth simulation.

We also compare the number of submissions required to achieve this credibility level when randomly selecting reviewers from the set $\mathcal{R}$ with a uniform probability, as described in the main text, to a more aggressive strategy where the author utilizes information from previous submissions to suggest reviewers. This aggressive strategy involves selecting reviewers, sampling $\mathcal{S}_m$, based on the marginal posterior of previous submissions — $\rho_i(m-1)$ as defined in Eq. 10 of the main text. The first suggested reviewer is selected with a probability proportional to their marginal posterior, and this process is repeated for the remaining reviewers, excluding those that have been previously selected.

The number of misclassifications done when the author stops at 95% credibility for the top three reviewers is presented in Fig. 1. This figure is based on the cynical model simulation presented in the main text (5 out of 10 friends in the simulation's ground truth). We observe that the set of 3 highest credibility reviewers has at least one misclassified reviewer in 6.9% of cases. We also present analogous results when using the aggressive strategy to obtain 95% credibility for the top three suggested reviewers in Fig. 1. It follows that although fewer submissions are necessary to obtain that credibility when using the aggressive strategy, it comes at a trade-off of more common misclassifications (8.3%).

Similar results for a ground truth with 9 friends are presented in Fig. 2. When compared to the aggressive strategy, the gain in the number of submissions is modest (both around 35 submissions and similar number of mistakes). The results for the quality model with 5 friends are presented in Fig. 3. In that case, the difference in the number of submissions necessary is visible (mean value of approximately 300 in the aggressive strategy against 400 submissions) and the number of misclassification decreases (from 8.9% to 7.2%). The number of submissions, however, is still too large to identify a group of friendly reviewers for most researchers. In the quality model with 9 friends, presented in Fig. 4, a pattern similar to the cynical model emerges: the gain in the number of submissions is modest, but misclassifications increase from 3.0% to 3.2%.

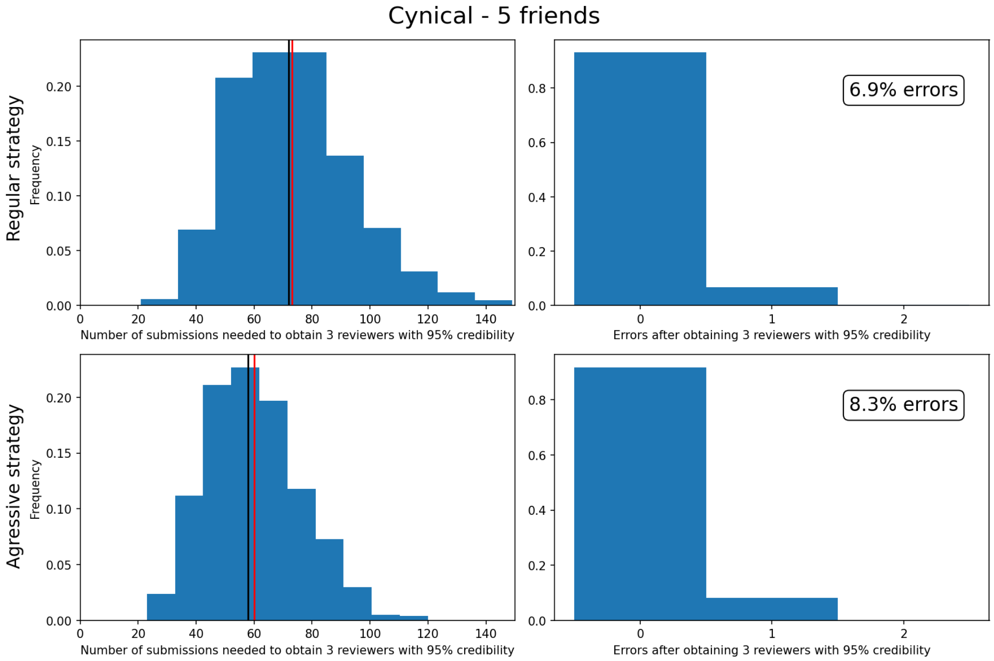

Fig 1. Number of submissions necessary to obtain 95% credibility in the three most likely reviewers to be friendly (on the left) and number of misclassified reviewers among these three (on the right). These results are for the cynical model simulation with 5 (out of 10) friends in the simulation's ground truth. When comparing the regular (uniform) strategy to the aggressive strategy, the number of submissions has a modest decrease. Meanwhile, the probability of having at least one misclassification increases.

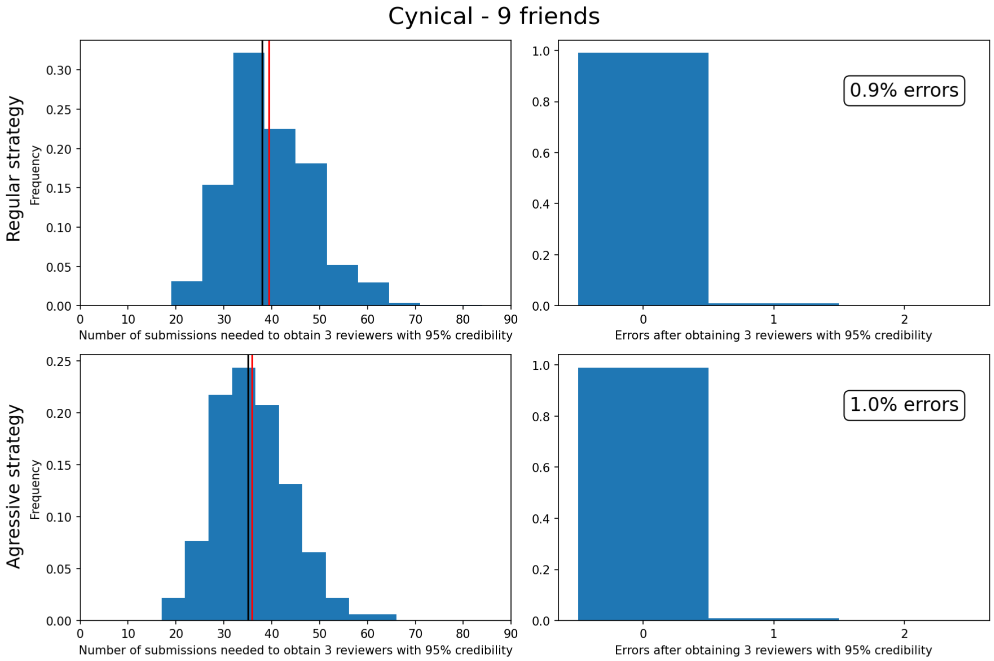

Fig 2. Number of submissions necessary to obtain 95% credibility in the three most likely reviewers to be friendly (on the left) and number of misclassified reviewers among these three (on the right). Made for the cynical model and 9 (out of 10) friends in the simulation's ground truth. When switching from the regular to the aggressive strategy, the number of submissions had a modest decrease.

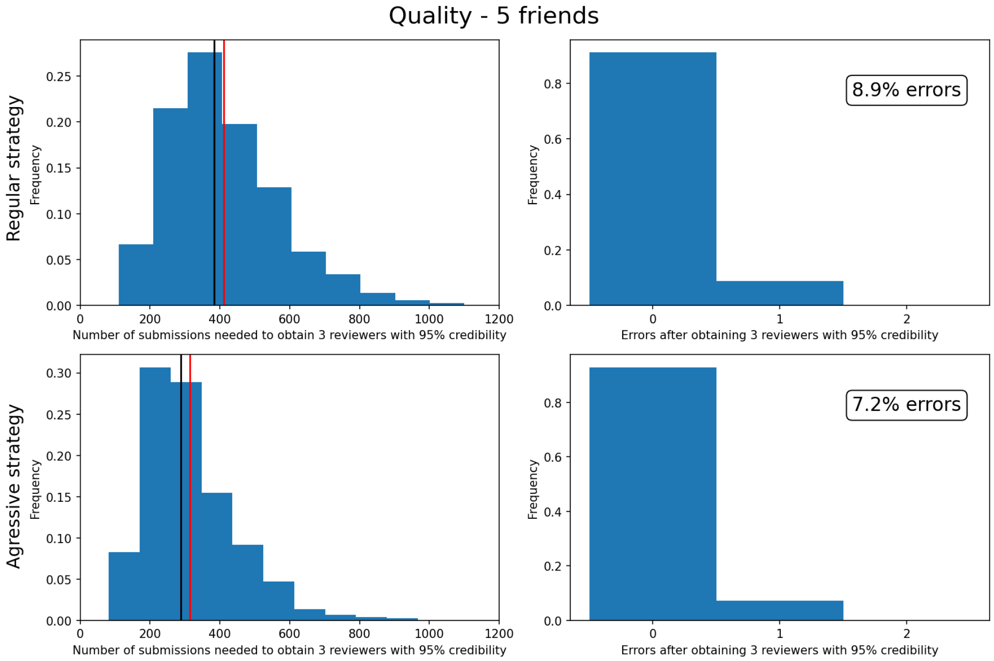

Fig 3. Number of submissions necessary to obtain 95% credibility in the three most likely reviewers to be friendly (on the left) and number of misclassified reviewers among these three (on the right). These results are for the quality model simulation with 5 (out of 10) friends in the simulation's ground truth. When switching from the regular to the aggressive strategy, the number of submissions had a significant decrease while the number of misclassifications descreased.

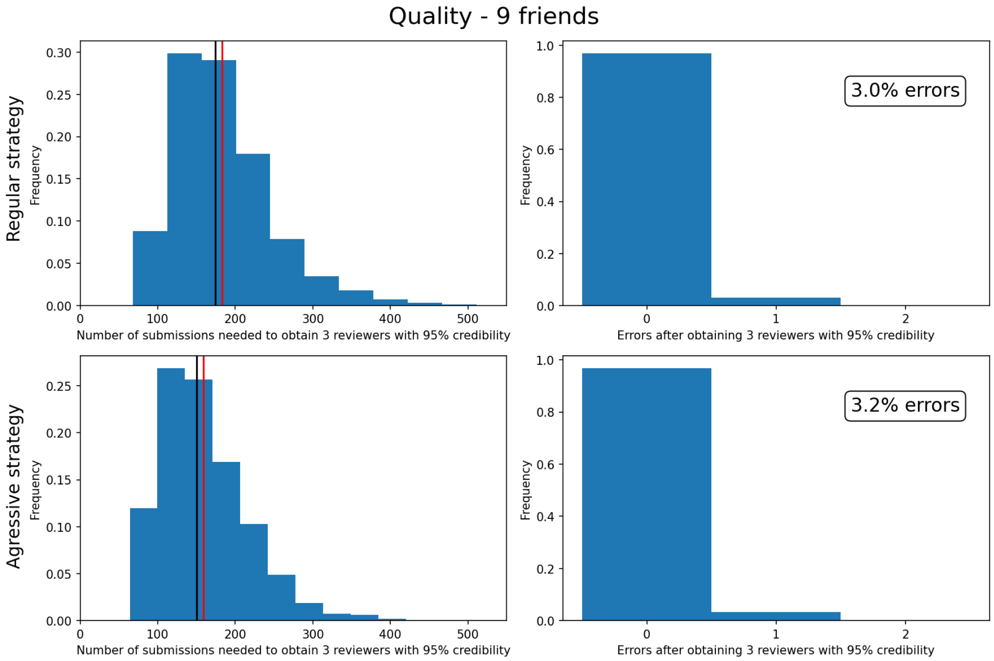

Fig 4. Number of submissions necessary to obtain 95% credibility in the three most likely reviewers to be friendly (on the left) and number of misclassified reviewers among these three (on the right). These results are for the quality model simulation with 9 (out of 10) friends in the simulation's ground truth. When switching from the regular to the aggressive strategy, the number of submissions had a modest decrease.
