# Supplementary material for: How many submissions are needed to discover friendly suggested reviewers?
Source: PLoS One. 2023 Apr 13;18(4):e0284212. doi: 10.1371/journal.pone.0284212 (PMC10101443; doi:10.1371/journal.pone.0284212)
Supplement: Attachment — Submitted filename: Reply.pdf [file pone.0284212.s006.pdf]

Reply to Reviewer reports – PONE-D-23-03685

We are thankful for the editorial efforts and reviewers suggestions in our manuscript. Overall, both reviewers have indicated agreement with publication. In what follows we answer to give a point-by-point answer to each of their queries. Changes in the manuscript are marked in red.

Reviewer #1:

> In this work the authors combined agent-based models to emulate single-blind peer review processes and a Bayesian inference system to set a lower bound in terms of how many papers are necessary to an author to determine the "friendliness" of a reviewer. The results presented show that even in the most simple scenarios, the lower bound is too high for non very prolific authors.
>
> The paper is well written in English. I suggest to accept this paper after some minor revisions suggested below.

We thank this referee and refer them to our responses below.

> line 46, I think that the term "unfeasibly" should be changed into "unfeasible".

Thanks! We made the edit.

> I don't think that a new paragraph is required at lines 84/85.

Paragraphs have now been combined.

> line 96, the authors forgot to put a "the" before easiest.

Fixed!

> I don't know if such a problem is due to the submission process or intended by the authors, but in the latter case I suggest them to remove the red squares around the hyper-text links.

It's a Latex feature and we have kept it for now.

> line 203, the authors state "for a fixed a data set", the 'a' after 'fixed' is not necessary.

Fixed!

> I suggest to the authors to use vector images, such as pdf images, to strongly improve the readability of the figures and the overall quality of the paper.

This was an oversight from lack of familiarity with PLOS submission system. We apologise and the current version has images in pdf format.

> In this work, the authors leveraged simple models and already obtained sound lower bounds. Nonetheless, I think it would be interesting to discuss how the lower bounds increase if even more complicated models are considered. I'm aware that this request can be out of the scope of the paper, but models characterized by more realistic rules and numbers can be of interest and give more realistic bounds. I suggest them to evaluate how the boundaries change if an author can suggest more than 3 reviewers and if a large number of impartial reviewers are considered; i.e., the set R is larger.

We thank the referee for this helpful suggestion. We have re-run the simulation for authors suggesting 4 or 5 reviewers. These are part of the new SI 1 (the other SI were renumbered). The results are as expected, they raise the number of submissions needed.

In our SI we tried to access a comprehensive array of relevant cases for reliability analysis. However if one desires to see the results of other simulations,(*e.g* with other values for the number of suggested reviewers or larger pool of reviewers) they can check the documented code in our GitHub repository [`https://github.com/PessoaP/how_many_submissions`].

Reviewer #2:

> This is a very interesting and, I believe, novel exercise to ascertain how easy it is for authors to influence the publication outcome of their articles by suggesting potentially 'friendly' reviewers. Reassuringly, the answer based on the agent-based model used here is that it is extremely difficult. As the authors state, 'the single-blind review process is sufficiently reliable to allow authors to suggest their own reviewers without clouding or biasing the publication decision'.
>
> Being a non-expert in agent-based modelling, I am not qualified to comment on the methodology used or the accuracy of the interpretation of the results. Actually this may not be too problematic, because many people for whom this work will be of interest may also lack training in ABM, and the authors might consider elaborating some concepts to help understanding. If any of my comments below betray my lack of expertise in ABM, I hope the authors will make allowance for this.

We thank the referee for their time and comments. Please see our responses below.

> That aside, I do have a few observations and questions relating to the broader questions behind the authors' work:
>
> They state that one of the challenges in exploring whether reviewers are positively or negatively inclined is that manuscript history and reviews are 'kept under lock and key'. This is not correct; some of the studies they cite are based on actual journal data. Moreover, the PEERE group assembled a vast data set that, although now a few years old, could be used to explore the question (`https://dataverse.harvard.edu/dataset.xhtml?persistentId=doi:10.7910/DVN/3IKRGI`). If the authors wish to comment in their Discussion on future possible avenues of study, they could encourage others to apply some of their methods to real data sets.

We thank the referee for pointing this out. We changed the abstract and the first paragraph of the conclusion (changes starting at line 307) to take into account the reviewer's suggestion. In it we

mention that the this peer-review data is still insufficient to answer the title question because it is anonymized.

> The modelling is predicated on a binary 'accept' – 'reject' inclination on the part of reviewers. In practice there is more of a spectrum from positive to negative sentiment on the part of reviewers about a manuscript. This affects the applicability of the ABM and is a limitation that I don't believe the authors commented on, unless I missed it.

This is an important point that we have alluded to but failed to make clear. The notion of the spectrum in editorial responses, and any other realistic feature, only makes the assessment of who is a friendly reviewer ever more difficult. We introduce new text on both the methods (starting at line 74) and Discussion (line 331) sections to clarify the point.

> Authors may submit the same article to multiple journals. How much would this affect the outcome of the model used by the authors? 'Submission' and 'manuscript' are often used interchangeably, but it seems important to distinguish here between the actual manuscript and the act of submission. Moreover, different journals may set different expectations for reviewers even for the same manuscript – for example, reviewers for a journal that looks for novelty may evaluate the same manuscript differently from how they would evaluate it for a journal that looks only for sound science. This is another layer of stochasticity that should be mentioned.

This is an excellent point now highlighted in the Discussion (new paragraph starting at line 337).

> Could another layer of stochasticity be that a niche field will have a much smaller pool of reviewers, and possibly also reviewers may be either much more positively inclined towards the author's work – because they know the author well – or much more negatively inclined towards it – because it competes directly with their own?

This is another excellent point that we expand upon on the Discussion (starting at line 351).

> I am not sure I understand why, in the model, authors have access only to the number of positive reviewers, and not also to the number of negative reviews.

We apologize for the confusion. The authors have access to the reviewer reports, which are classified as positive or negative. It is not that the author only has access only to the number of positive reviews, but rather that the number of positive reviews has all the information gathered, since the number of negative reviews is the total number positive reviews subtracted from the total number of reports (which is always 2 in our model). In order to avoid this confusion, we changed the bullet point 4) in our Methods section (line 61).

> How would the results of the model be affected if potentially friendly reviewers were to decline to review (because, for example, they realise they have a conflict of interest)?

That's an excellent point. We discuss on the second to last paragraph of the conclusion (line 356) how this makes the suggestion of reviewers with whom the authors have close relationship less useful to the editor.
